# Genetic Dissection of Plant Height Variation Between the Parental Lines of the Elite *Japonica* Hybrid Rice ‘Shenyou 26’

**DOI:** 10.3390/ijms262010155

**Published:** 2025-10-18

**Authors:** Bin Sun, Xiaorui Ding, Kaizhen Xie, Xueqing Zhang, Can Cheng, Yuting Dai, Anpeng Zhang, Jihua Zhou, Fuan Niu, Rongjian Tu, Yue Qiu, Zhizun Feng, Bilian Hu, Chenbing Shao, Hongyu Li, Tianxing Shen, Liming Cao, Huangwei Chu

**Affiliations:** 1 Key Laboratory of Germplasm Innovation and Genetic Improvement of Grain and Oil Crops (Co-Construction by Ministry and Province), Ministry of Agriculture and Rural Affairs, Crop Breeding and Cultivation Research Institute, Shanghai Academy of Agricultural Sciences, Shanghai 201403, China; sxb0708@126.com (B.S.); chengcan@saas.sh.cn (C.C.); zhanganpeng@saas.sh.cn (A.Z.); qiuyue0124@outlook.com (Y.Q.);; 2 Shanghai Key Laboratory of Agricultural Genetics and Breeding, Shanghai Academy of Agricultural Sciences, Shanghai 201106, China

**Keywords:** rice, plant height, quantitative trait loci, breeding, semi-dwarf

## Abstract

Plant height is a key agronomic trait influencing both seed production and yield in hybrid rice. In the elite japonica hybrid ‘Shenyou 26’, optimal plant height differences between the restorer line (‘Shenhui 26’) and the male sterile line (‘Shen 9A’) are critical for efficient pollination. In this study, we dissected the genetic basis of plant height variation using a doubled haploid (DH) population derived from ‘Shenyou 26’. Multi-environment phenotyping and QTL mapping identified seven QTLs associated with plant height, among which *qPH1.1* and *qPH9.1* were validated. *qPH1.1* co-localized with the semi-dwarf gene *SD1*, and ‘Shen 9A’ carries a rare *SD1-EQH* allele that potentially confers reduced height relative to the *SD1-EQ* allele in ‘Shenhui 26’. *qPH9.1* also contributed significantly to plant height variation, with the Shenhui26 allele increasing plant height in backcross validation. These findings indicate that plant height variation in ‘Shenyou 26’ is controlled by multiple loci, including *SD1* allelic variants and other complementary QTLs, providing valuable resources for fine-tuning plant architecture in rice breeding.

## 1. Introduction

Plant height is a pivotal agronomic trait that strongly influences rice yield. Excessive plant height increases the risk of lodging and subsequent yield losses, whereas overly short stature can restrict yield potential by limiting biomass accumulation. Thus, optimizing plant height to balance lodging resistance with sufficient biomass production remains a central goal in modern rice breeding [1].

Since the 1960s, the introduction of the *semi-dwarf 1* (*sd1*) gene from the rice variety Dee-Geo-Woo-Gen (DGWG) has dramatically increased rice yields, establishing a cornerstone of the “Green Revolution” in rice breeding [2]. By reducing plant height, *sd1* enhanced the harvest index and resolved the trade-off between high yield potential and lodging resistance under high nitrogen fertilizer input [3,4]. Notably, the *SD1* locus had already undergone artificial selection prior to the “Green Revolution”. During *japonica* domestication, two functional nucleotide polymorphisms (FNPs) arose in the *japonica SD1* allele (*SD1-EQ*), reducing GA20ox2 enzyme activity, lowering endogenous gibberellin (GA) levels, and thereby decreasing plant stature. In contrast, most *indica* landraces and the wild progenitor *O. rufipogon* retain the strongly functional *SD1* allele (*SD1-GR*), which confers higher GA content and taller growth [5,6,7]. At present, *SD1* remains one of the most widely exploited genes in rice improvement, largely due to its abundant natural polymorphism [8,9,10].

However, the widespread deployment of *SD1* mutant alleles in rice has also generated unintended consequences. For instance, sustaining high yields in these varieties often requires intensive nitrogen fertilization, which contributes to eutrophication of aquatic ecosystems [11,12]. Moreover, the overreliance on the *SD1* mutant alleles not only narrows genetic diversity but also introduces unfavorable traits, including reduced spikelet fertility under low-temperature conditions and heightened sensitivity to drought stress [13,14,15]. To mitigate the drawbacks of excessive reliance on *sd1*, diversifying the genetic resources for semi-dwarfism through the identification and use of novel, practically valuable genes is essential for sustaining and strengthening the genetic resilience of future rice breeding programs [16,17].

Rice plant height is a complex trait governed by both qualitative and quantitative genes. To date, more than 1000 QTLs associated with plant height have been mapped, and dozens of genes related to dwarfism or semi-dwarfism genes have also been identified from mutants [4,18,19]. However, most of these genes are unsuitable for breeding because they confer undesirable agronomic traits such as excessive dwarfism, reduced fertility, or short grains. Therefore, identifying novel favorable alleles that achieve an optimal balance between plant height and yield remains essential for future rice improvement [20].

In this study, a doubled haploid (DH) population derived from the elite japonica hybrid rice ‘Shenyou 26’ was used to identify QTLs associated with plant height. A total of seven QTLs were detected, including *qPH1.1*, which may be associated with the *SD1* locus, while the genetic effect of *qPH9.1* was further validated. These findings contribute to elucidating the genes underlying plant height regulation and provide valuable genetic resources for fine-tuning plant height in rice breeding.

## 2. Results

### 2.1. Height Variation Between the Male-Sterile (Shen 9A) and Restorer (Shenhui 26) Lines in the ‘Shenyou 26’ Hybrid System

**Figure 1:**
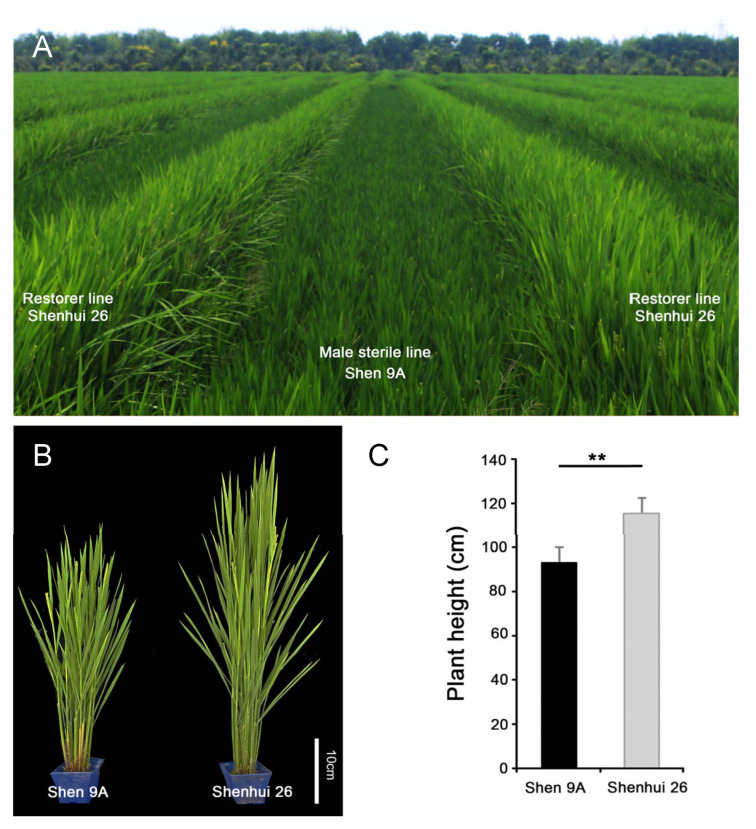
Comparison of the male sterile line “Shen 9A” and restorer line “Shenhui26”. (**A**) Field performance of “Shen 9A” and “Shenhui26” at the early heading stage in the hybrid seed production field. (**B**) Plant architecture of “Shen 9A” and “Shenhui26” at the early heading stage. (**C**) Plant height of “Shen 9A” and “Shenhui26”, Data are presented as mean ± SD (*n* = 20); ** *p* ≤ 0.01.

The three-line japonica hybrid rice ‘Shenyou 26’ was developed by the Shanghai Academy of Agricultural Sciences (SAAS) through using ‘Shen 9A’, a BT-type cytoplasmic male sterile (CMS) line, as the female parent, and the restorer line ‘Shenhui 26’ as the pollen donor [21]. At the heading stage, ‘Shenhui 26’ exhibits slightly taller than ‘Shen 9A’ (Figure 1A–C), ensuring optimal pollination efficiency—a critical factor for high hybrid seed yield. However, the genetic basis underlying this plant height difference remains unclear.

### 2.2. Genetic Basis of Plant Height Differentiation Between ‘Shen 9A’ and ‘Shenhui 26’

**Figure 2:**
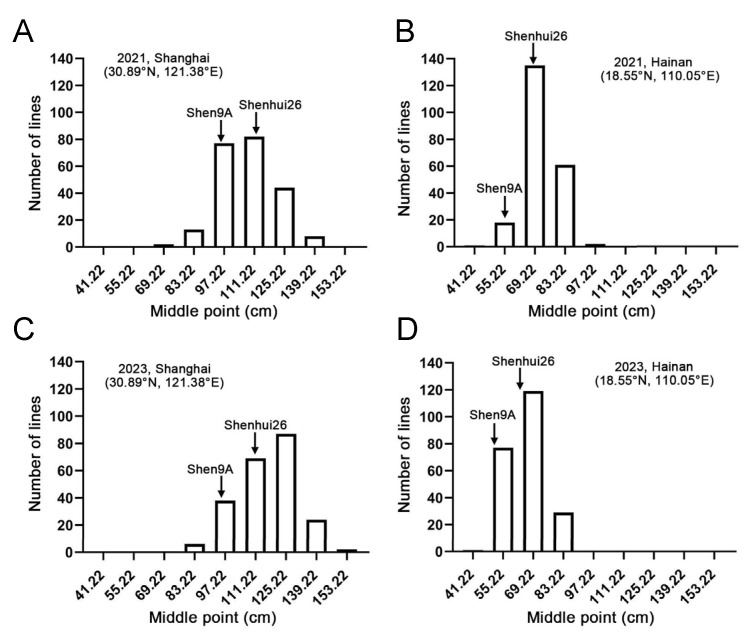
Frequency distribution of plant height in the DH population grown in Shanghai in 2021 (**A**) and 2023 (**C**) and in Hainan in 2021 (**B**) and 2023 (**D**).

Previously, a doubled haploid (DH) population comprising 232 lines was constructed using young panicles of ‘Shenyou 26’ through anther culture [22]. To investigate the genetic basis of plant height differentiation between ‘Shen 9A’ and ‘Shenhui 26’, plant height was measured and analyzed for the DH population and its parental lines when grown in Shanghai (30.89° N, 121.38° E) and Hainan (18.55° N, 110.05° E) during the 2021 and 2023 growing seasons. Under all four environmental conditions, plant height in the DH population exhibited a continuous variation that approximated a normal distribution. Furthermore, transgressive segregation in both directions was observed (Table 1; Figure 2A–D), indicating that the plant height differentiation between ‘Shen 9A’ and ‘Shenhui 26’ is controlled by multiple quantitative trait loci. Notably, mean plant height for both the DH and parental lines was significantly greater in Shanghai (30.89° N, 121.38° E) than in Hainan (18.55° N, 110.05° E), a trend consistent across both growing seasons (Table 1). These results underscore the profound impact of environmental conditions on rice plant height. Indeed, this environmental responsiveness is characteristic of ‘Shenyou 26’, a photoperiod-sensitive japonica hybrid particularly well-adapted to Shanghai and similar latitudes.

ANOVA analysis was performed to elucidate the sources of plant height variation in the DH population. The resulting variance components and broad-sense heritability (*H*^2^) are summarized in Table 2. The analysis revealed that the environmental variance (*σ*^2^*_E_* = 662.36) was the largest component and substantially greater than the genotypic variance (*σ*^2^*_G_* = 86.01) and the genotype-by-environment interaction variance (*σ*^2^*_GE_* = 48.21), indicating that environmental variance constitutes the predominant proportion of the total phenotypic variation. This result further reinforces the profound influence of environmental conditions on rice plant height.

Despite the substantial environmental influence, the broad-sense heritability (*H*^2^) for plant height was 0.88 (Table 2). This high heritability of plant height indicates that, within a given environment, the observed phenotypic variation among the parental and DH lines is largely attributable to genetic factors, confirming that plant height is a highly heritable trait in this population. This conclusion is also supported by the consistently greater plant height of ‘Shenhui 26’ compared to ‘Shen 9A’ in both Shanghai (30.89° N, 121.38° E) and Hainan (18.55° N, 110.05° E) across two growing seasons (Table 1).

### 2.3. QTL Identified for the Plant Height in the DH Population

Based on a previously constructed genetic linkage map comprising 470 SNP markers for this DH population [22], a QTL analysis was conducted to identify genomic regions associated with plant height. An LOD threshold of 3.0, determined by 1000 permutations, was applied to declare significant QTLs. A total of seven QTLs were identified on chromosomes 1, 2, 5, 6, 9, and 12 (Table 3). Among these, *qPH1.1* was consistently detected across all four environments, while *qPH5.1* and *qPH9.1* were identified in three environments. The remaining four QTLs were environment-specific, each detected in only one environment. The QTLs *qPH1.1*, *qPH9.1*, and *qPH9.2* exhibited positive additive effects, indicating that the allele from ‘Shenhui 26’ contributed to increased plant height.

### 2.4. The SD1 Gene Is a Probable Candidate for the qPH1.1

The stable QTL *qPH1.1*, which was consistently detected across all four environments, was mapped to a region on chromosome 1 that is proximal to the semi-dwarf gene *SD1*, which is widely recognized as a cornerstone of the ‘Green Revolution’ in rice breeding due to its crucial role in determining plant height. A pronounced reduction in nucleotide diversity has been observed around the *SD1* locus in *japonica* landraces, a pattern not detected in *indica* landraces or *O. rufipogon*, indicating that the *SD1* gene underwent strong artificial selection during *japonica* domestication [5]. Compared to the *indica* variety ‘Kasalath’, two single-base mutations in the *SD1* gene of the *japonica* cultivar ‘Nipponbare’ lead to two amino acid substitutions (G100E and R340Q) in the encoded GA20 oxidase, which significantly reduces plant height [5].

**Figure 3:**
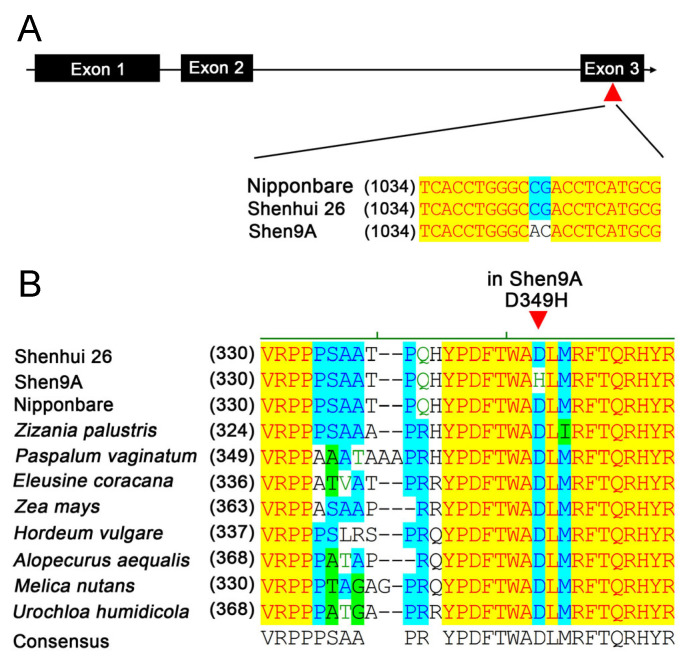
Sequence analysis of the *SD1* gene and its encoded GA20 oxidase. (**A**) Gene structure of *SD1* and alignment of coding sequences. (**B**) Comparison of GA20 oxidase amino acid sequences among ‘Shenhui 26’, ‘Shen 9A’, and other *Poaceae* species.

Sequence analysis revealed that the restorer line ‘Shenhui 26’ carries the *SD1-EQ* allele, identical to that reported in ‘Nipponbare’ [5]. In contrast, the male sterile line ‘Shen 9A’ harbors both the characteristic *SD1-EQ* mutations and two additional nucleotide substitutions in exon 3 (Figure 3A), resulting in an amino acid change (D349H). Further analysis identified that *SD1-EQH* in ‘Shen 9A’ is identical to the *sd1-r* allele derived from ‘Reimei’ [8]. Multiple sequence alignment revealed that the aspartic acid at position 349 is highly conserved in GA20 oxidase across diverse *Poaceae* species (Figure 3B), suggesting that this residue is likely required for the full activity of GA20 oxidase. Therefore, the D349H substitution may impair enzyme function, thereby contributing to the plant height variation observed in ‘Shen 9A’.

**Figure 4:**
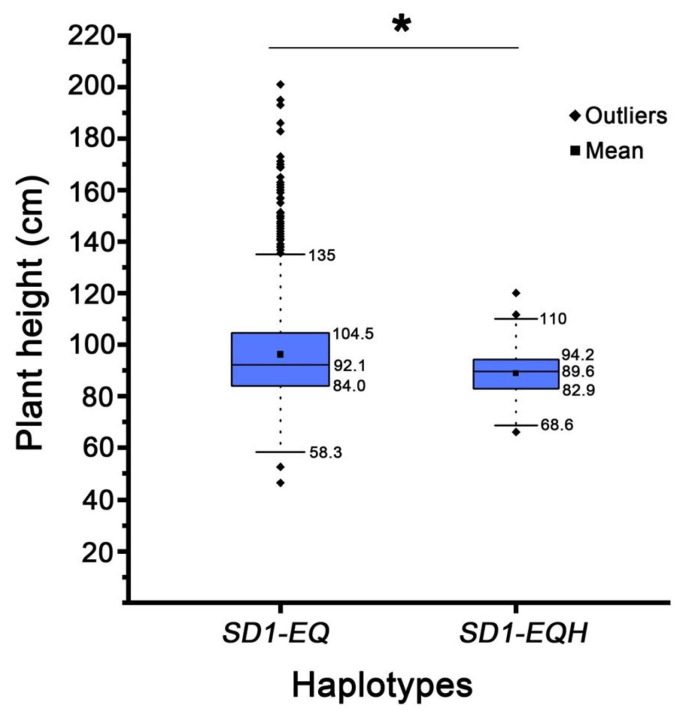
Box plot of plant height of *SD1-EQ* and *SD1-EQH* haplotypes in rice, * *p* ≤ 0.05.

A haplotype analysis of the *SD1* gene was performed using the MBKbase database (www.mbkbase.org) [23]. The results revealed that 1343 accessions carried the *SD1-EQ* allele, which is identical to that in ‘Shenhui 26’, while only 82 accessions possessed the *SD1-EQH* allele matching that of ‘Shen 9A’. Comparative analysis of plant height between accessions with these two haplotypes showed that those carrying the *SD1-EQH* allele were significantly shorter than those with the *SD1-EQH* allele (*p* < 0.05) (Figure 4), indicating that the D349H substitution may impair the biochemical function of the protein. Taken together, these results all support *SD1* as the causal gene underlying the *qPH1.1* locus. Furthermore, the *SD1-EQH* allele represents a valuable genetic resource for fine-tuning plant height in *japonica* rice breeding programs.

### 2.5. Genetic Validation for the qPH9.1 Locus

**Figure 5:**
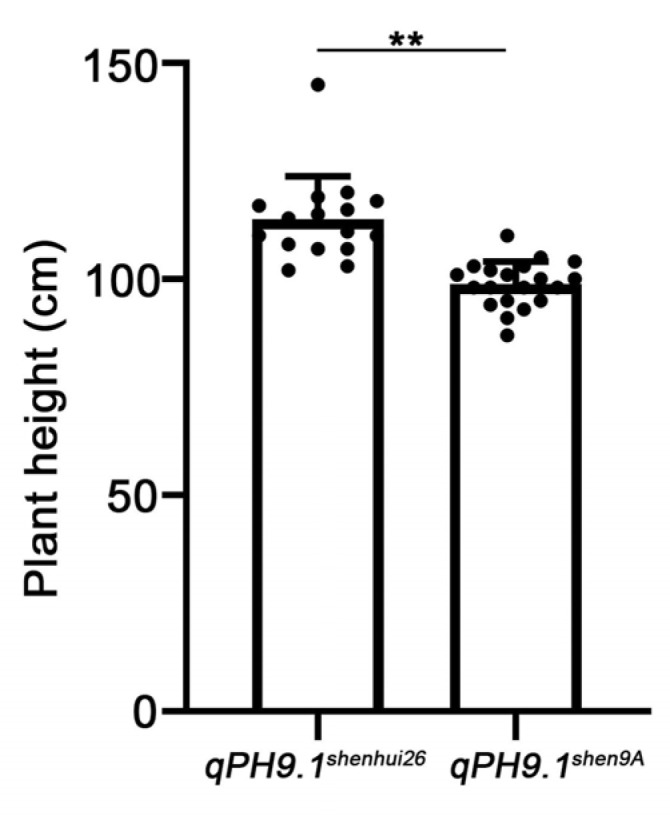
Plant height comparison between lines carrying the *qPH9.1^Shenhui26^* allele and the *qPH9.1^Shen9A^* allele in the BC_1_F_2_ population, ** *p* ≤ 0.01.

The QTL mapping results revealed that the *qPH9.1* locus was consistently detected in three environments (2021 Shanghai, 2021 Hainan, and 2023 Hainan), explaining a considerable proportion of phenotypic variance with contribution rates of 7.42%, 22.72%, and 8.36%, respectively (Table 3). To further validate the effect of the *qPH9.1* locus, two flanking InDel markers, C9-16503961 and C9-17927666 (Table S1, Figure S1), were developed to genotype a BC_1_F_2_ population consisting of 96 lines derived from a backcross with ‘Shen 9A’ as the recurrent parent. Among these lines, 17 carried the *qPH9.1^Shenhui26^* allele and 21 carried the *qPH9.1^Shen9A^* allele. Phenotypic comparison showed that lines with the *qPH9.1^Shenhui26^* allele were significantly taller than those with the *qPH9.1^Shen9A^* allele (Figure 5). Although minor background effects could not be completely excluded, the significant difference in mean plant height between the two genotypes strongly supports the conclusion that the *qPH9.1^Shenhui26^* allele positively regulates plant height. These results confirm that the *qPH9.1* locus has potential value for modifying plant height in rice breeding.

## 3. Discussion

Hybrid rice has made substantial contributions to global food security [24]. The elite *japonica* three-line hybrid rice ‘Shenyou 26’, developed by the Shanghai Academy of Agricultural Sciences, was derived from a cross between the BT-type male sterile line ‘Shen 9A’ and the restorer line ‘Shenhui 26’. The hybrid was approved by the Shanghai Crop Variety Approval Committee in 2017 and shows good adaptability in the suburban areas of Shanghai, southern Jiangsu, northern Zhejiang, and southern Anhui provinces [21]. From 2019 to 2021, yield evaluations conducted across ten seed production bases in the suburban areas of Shanghai, covering a total of 3334 mu of ‘Shenyou 26’ seed production fields, showed an average seed yield of 245.5 kg per mu. One important factor influencing successful seed production in ‘Shenyou 26’ seed production is the plant height relationship between parental lines. Specifically, having the restorer line ‘Shenhui 26’ slightly taller than the male sterile line ‘Shen9A’ promotes efficient pollination and maximizes seed yield (Figure 1).

In this study, we investigated the genetic basis underlying variation in plant height between the A-line ‘Shen 9A’ and the R-line ‘Shenhui 26’ in the elite *japonica* hybrid ‘Shenyou 26’. Through multi-environment phenotyping and QTL mapping, we identified seven QTLs associated with plant height. These findings not only deepen our understanding of the genetic architecture of plant height in *japonica* rice but also provide practically valuable alleles for optimizing plant height in rice breeding.

The QTL *qPH1.1*, stably detected across all environments, was mapped to the genomic region containing the semi-dwarf gene *SD1*. Sequence comparison revealed that ‘Shenhui 26’ carries the *SD1-EQ* allele [5], whereas ‘Shen 9A’ harbors a different allele (*SD1-EQH/sd1-r*) with a D349H substitution in GA20 oxidase [8,25]. This substitution affects a highly conserved residue across grasses (Figure 3B) and may reduce enzymatic activity, potentially contributing to the reduced height of ‘Shen 9A’. Haplotype analysis in a large germplasm panel further demonstrated that accessions with *SD1-EQH* were significantly shorter than those carrying *SD1-EQ* (Figure 4), providing further evidence that *SD1* is the causal gene underlying *qPH1.1*. Recent research showed that replacing the non-functional *SD1* allele in the indica cultivar ‘9311’ with the weak allele *SD1-EQ* led not only to a substantial increase in plant height but also to an increase in yield per plant [7]. Thus, the *SD1-EQ* and *SD1-EQH* alleles represent variants with different strengths and may serve as valuable genetic resources for fine-tuning plant height in rice breeding, as they confer different levels of height reduction.

In addition to *qPH1.1*, we validated the genetic effect of *qPH9.1*, which explained 7.4181% to 22.7% of the phenotypic variance in various environments. Backcross validation demonstrated that the *qPH9.1^Shenhui26^* allele increases plant height relative to the *qPH9.1^Shen9A^* allele. The identification of *qPH9.1* complements the long-recognized contribution of *SD1*, indicating that multiple loci contribute to plant height variation in the ‘Shenyou 26’-derived DH population. Such QTLs provide promising new targets for marker-assisted selection and may help overcome the drawbacks of overreliance on *sd1* in rice plant height manipulation.

Our analysis also underscored the profound influence of the environment on plant height expression. Although broad-sense heritability was high (*H*^2^ = 0.88), environmental variance accounted for the largest proportion of phenotypic variation. Consistent with the photoperiod sensitivity of ‘Shenyou 26’, plants grown in Shanghai were significantly taller than those in Hainan across both years (Table 1). These results highlight the importance of considering genotype–environment interactions when manipulating plant height in rice breeding. In particular, when developing varieties adapted to different ecological regions, it is crucial to select appropriate semi-dwarf alleles or plant height-related genetic resources that match the specific environmental conditions of each region.

Together, our results reveal that plant height variation between ‘Shen 9A’ and ‘Shenhui 26’ is influenced by multiple loci, including *qPH1.1* (*SD1*) and *qPH9.1*. The identification of multiple allelic variants offers new opportunities for fine-tuning plant architecture in rice breeding. Incorporating these loci into breeding programs may help balance plant height and yield potential, reduce lodging risk, and improve pollination efficiency in hybrid seed production. Future work focusing on the cloning and functional characterization of *qPH9.1*, as well as exploring its interaction with other height-related genes, will further enhance our understanding of plant height regulation and facilitate the development of high-yielding, environmentally adaptable rice varieties.

## 4. Materials and Methods

### 4.1. Plant Materials and Field Trials

A doubled haploid (DH) population comprising 232 individual lines derived from the elite japonica hybrid rice ‘Shenyou 26’ was used to map QTLs associated with plant height regulation [22]. ‘Shenyou 26’ was developed from a cross between the BT-type cytoplasmic male sterile (CMS) line ‘Shen 9A’ (used as the female parent) and the restorer line ‘Shenhui 26’ (used as the pollen donor) [21].

The DH population lines and their parental lines were cultivated from May to November in Shanghai (30.89° N, 121.38° E) and from December to April of the following year in Hainan (18.55° N, 110.05° E), China. A randomized complete block design with two replications was implemented at each location. Each plot consisted of six rows, with six plants per row. Field management practices during the growing season followed local farmers’ standard methods. Plant height was measured from ten representative plants located in the central area of each plot, with height defined as the distance from the soil surface to the top of the panicle.

### 4.2. ANOVA Analysis

Analysis of variance (ANOVA) was conducted using QTL IciMapping software [26]. The linear model used for ANOVA followed the description of Yin et al. [27]:

(1) $$y_{ijk}=\mu +R_{k/j}+G_{i}+E_{j}+{\left(GE\right)}_{ij}+\varepsilon_{ijk},\;{\;\;\varepsilon }_{ijk}\sim N\left(0,\;\sigma_{\varepsilon }^{2}\right)$$

where *y_ijk_* is the phenotypic value of the *i*-th (*i* = 1~232) genotype in the *j*-th (*j* = 1~4) environment and the *k*-th (*k* = 1∼2) replication, *μ* is the overall mean of the DH population, *R_k/j_* is the replication effect within environment *j*, *G_i_* is the genotypic effect, *E_j_* is the environmental effect, (*GE*)*_ij_* is the genotype-by-environment interaction, and *ε_ijk_* is the random error.

Variance components, including genotypic variance (*σ*^2^*_G_*), environmental variance (*σ*^2^*_E_*), genotype-by-environment interaction variance (*σ*^2^*_G×E_*), and error variance (*σ*^2^*ε*), were estimated from the ANOVA results. Broad-sense heritability (*H*^2^) was calculated as:

(2) $$H^{2}=\frac{\sigma_{G}^{2}}{\sigma_{G}^{2}+\frac{1}{e}\sigma_{G\times E}^{2}+\frac{1}{er}\sigma_{\varepsilon }^{2}}$$

where *e* is the number of environments and *r* is the number of replications [27].

### 4.3. QTL Mapping

A genetic linkage map, previously constructed [22], was used for QTL mapping. QTLs associated with rice plant height were identified using Inclusive Composite Interval Mapping (ICIM) implemented in QTL IciMapping software [26]. To determine significant QTLs, an LOD threshold of 3 was applied, which was estimated from 1000 permutations at a genome-wide type I error rate of 0.05. The phenotypic variance explained (PVE) and additive effects of each detected QTL were also calculated to evaluate their contribution to plant height variation. The confidence interval for each QTL was defined as the genomic region corresponding to a 1.5-LOD drop from the peak LOD score, which approximates a 95% confidence interval. QTLs detected in different environments were considered to represent the same locus if their confidence intervals overlapped.

### 4.4. Validation for the qPH9.1 Locus

To validate the genetic effects of *qPH9.1*, the hybrid rice ‘Shenyou 26’ was backcrossed with the A-line ‘Shen 9A’. The resultant BC_1_F_1_ plants were genotyped using the flanking InDel markers C9-16503961 and C9-17927666. Heterozygous plants at the *qPH9.1* locus were self-pollinated to produce BC_1_F_2_ seeds. In 2025, the BC_1_F_2_ population was planted in Shanghai (30.89° N, 121.38° E) and genotyped with the same InDel markers. Plants homozygous for either *qPH9.1^Shenhui26^* or *qPH9.1^Shen9A^* were selected, and their plant heights were measured to compare phenotypic differences between the two genotypes.

## 5. Conclusions

Plant height variation between the parental lines of ‘Shenyou 26’ is controlled by multiple loci, including *SD1* allelic variants and *qPH9.1*. The complementary effects of these alleles enable precise regulation of plant height, providing valuable genetic resources for optimizing plant architecture and balancing the lodging resistance with yield in rice breeding.

## Figures and Tables

**Table 1:** Statistics of plant height of the parents and DH population in 4 environments.

Environment ^1^	Parents (cm)		DH Population			
	Shen9A	Shenhui26	Mean (cm)	Range (cm)	Skewness	Kurtosis
21SH	96.59	113.93	109.38	74.5–143.0	0.07	−0.17
21HN	55.47	67.90	72.40	46.5–98.5	0.16	0.32
23SH	93.16	110.36	117.93	84.5–150.5	−0.15	−0.46
23HN	50.37	62.27	66.74	44.0–90.0	0.23	−0.37

^1^ 21 and 23 refer to the years 2021 and 2023, respectively; SH and HN denote Shanghai (30.89° N, 121.38° E) and Hainan (18.55° N, 110.05° E), respectively.

**Table 2:** Variance components and heritability for the plant height estimated in the DH population.

Variance Components				Heritability (*H*^2^)
Environment (*σ*^2^*_E_*)	Genotype (*σ*^2^*_G_*)	G by E (*σ*^2^*_G×E_*)	Random Error (*σ*^2^*ε*)	
662.36	86.01	48.21	1.51	0.88

**Table 3:** QTLs of plant height identified in the four environments.

Environment	QTL	Chr.	Position ^1^	LOD	Flank Marker ^2^	PVE (%)	Add ^3^	CI (cM) ^4^
2021 Shanghai	*qPH1.1* *qPH5.1* *qPH9.1*	1 5 9	160 112 95	5.6490 6.9651 5.0192	Chr1-35346977~Chr1-37122278 Chr5-25700930~Chr5-26419104 Chr9-17208953~Chr9-17730888	8.4004 10.1566 7.4181	3.7806 −4.1561 3.577	154.5~161.5 110.5~113.5 92.5~100.5
2021 Hainan	*qPH1.1* *qPH2.1* *qPH5.1* *qPH9.1* *qPH12.1*	1 2 5 9 12	163 59 115 94 69	10.7983 3.1405 6.3262 19.0114 5.9827	Chr1-37122278~Chr1-38859748 Chr2-8900156~Chr2-8940541 Chr5-26419104~Chr5-27331103 Chr9-17208953~Chr9-17730888 Chr12-24973861~Chr12-25345406	12.0605 3.1628 6.7287 22.7247 6.2037	3.0255 −1.5828 −2.2572 4.1779 −2.1937	158.5~165.5 56.5~59.5 111.5~115.5 92.5~96.5 67.5~72.5
2023 Shanghai	*qPH1.1* *qPH5.1* *qPH9.2*	1 5 9	163 111 100	14.1927 8.4175 9.6674	Chr1-37122278~Chr1-38859748 Chr5-25616977~Chr5-25700930 Chr9-17730888~Chr9-18211888	18.6952 10.0683 11.7154	5.5662 −4.0886 4.4217	158.5~164.5 109.5~111.5 97.5~100.5
2023 Hainan	*qPH1.1* *qPH6.1* *qPH9.1*	1 6 9	163 137 93	11.4845 3.0276 5.6217	Chr1-37122278~Chr1-38859748 Chr6-29076596~Chr6-30825274 Chr9-17208953~Chr9-17730888	18.6777 4.4325 8.3606	3.7294 −1.8182 2.5112	158.5~165.5 135.5~142.5 91.5~96.5

^1^ Chromosome position (cM) of the LOD peak. ^2^ Flanking markers defining the interval containing the peak LOD. ^3^ A positive value indicates that the allele from ‘Shenhui 26’ increases plant height, while a negative value indicates that it decreases plant height. ^4^ Confidence interval (CI) represents a 95% probability range for the QTL location.

## Data Availability

Data are available upon request.

## Supplementary Materials

The following supporting information can be downloaded at https://www.mdpi.com/article/10.3390/ijms262010155/s1.
