# Supplementary material for: Genetic Dissection of Plant Height Variation Between the Parental Lines of the Elite Japonica Hybrid Rice ‘Shenyou 26’
Source: Int J Mol Sci. 2025 Oct 18;26(20):10155. doi: 10.3390/ijms262010155 (PMC12564361; doi:10.3390/ijms262010155)
Supplement: Supplementary file 1 [file ijms-26-10155-s001.zip › ijms-3922516-supplementary.pdf]

Supplementary Materials:

Table S1. Primer sequences of the flanking InDel markers developed for validating the *qPH9.1* locus.

Marker Name	Forward Frimer (5'-3')	Reversed Frimer (5'-3')	Amplified fragment Length (bp)
C9-16503961	GCCCTTCTATGTAGCACACC	GACATAACAAGGTGAGGAGGG	150 (Shen9A)/126 (Shenhui26)
C9-17927666	GCCAAATGCCAAATTACCAACT	GGGAACCGCTTCTAGCAAATC	144 (Shen9A)/133 (Shenhui26)

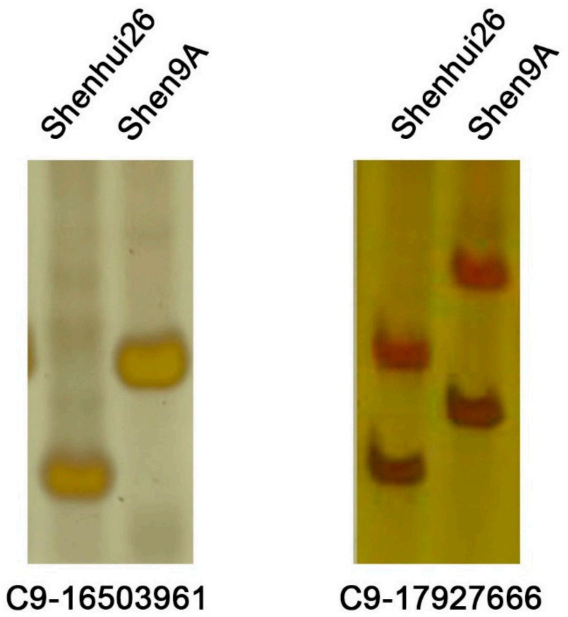

Figure S1. Electrophoresis showing polymorphism of the *qPH9.1* flanking InDel markers between the two parental lines.
